# Supplementary material for: Maternal folic acid supplementation and the risk of ankyloglossia (tongue-tie) in infants; a systematic review
Source: PLoS One. 2023 Nov 3;18(11):e0294042. doi: 10.1371/journal.pone.0294042 (PMC10624271; doi:10.1371/journal.pone.0294042)
Supplement: S1 Appendix — (DOCX) [file pone.0294042.s002.docx]

**Supporting Information**

**Maternal folic acid supplementation and the risk of ankyloglossia (tongue-tie) in infants; a systematic review**

Gal Rubin^1, 2^, Catherine Stewart^1^, Laura McGowan^3^, Jayne V Woodside^3^, Geraldine Barrett^1^, Keith M Godfrey^4^, Jennifer Hall^1^*

1. UCL EGA Institute for Women’s Health, Reproductive Health Research Dept, UCL, London
2. Minerva University: College of Natural Sciences, San Francisco, USA
3. Centre for Public Health, Institute for Global Food Security, Queen’s University Belfast, Belfast
4. MRC Lifecourse Epidemiology Centre and NIHR Southampton Biomedical Research Centre, University of Southampton and University Hospital Southampton NHS Foundation Trust

*Corresponding author: [jennifer.hall@ucl.ac.uk](mailto:jennifer.hall@ucl.ac.uk)

**Contents**

**Page 3 - S1 Appendix: Search Strategy**

**Page 4 - S1 Table: Excluded Articles**

**Page 12 - S2 Table:**

**Page 20 - S2 Appendix: PROSPERO Registration**

**S1 Appendix: Search Strategy**

| **Search Terms 1** |
| --- |
| Folic acid [MeSH Terms] OR Folic acid OR Folic acid supplementation OR Folic acid administration OR Folic acid adherence OR Folic acid fortification OR Folinic acid OR Serum folate OR Blood folate OR Plasma folate OR Homocysteine-lowering treatment OR B-Vitamins OR B vitamin complex |
| ***AND*** |
| Pregnant women OR Pregnancy OR Preconception OR Periconception |
| ***AND*** |
| Ankyloglossia [MeSH Terms] OR Ankyloglossia OR Tongue-tie OR Tongue tie |

Search Strategy 1

| **Search Terms 2** |
| --- |
| Folic acid [MeSH Terms] OR Folic acid OR Folic acid supplementation OR Folic acid administration OR Folic acid adherence OR Folic acid fortification OR Folinic acid OR Serum folate OR Blood folate OR Plasma folate OR Homocysteine-lowering treatment OR B-Vitamins OR B vitamin complex |
| ***AND*** |
| Pregnant women OR Pregnancy OR Preconception OR Periconception |
| ***AND*** |
| Frenotomy OR Lingual Frenum/surgery [MeSH Terms] |

Search Strategy 2

**S1 Table: Excluded Articles**

| Year | Author | Title | Journal |
| --- | --- | --- | --- |
| 2010 | Reamy, Brian V.; Derby, Richard; Bunt, Christopher W. | Common tongue conditions in primary care | American Family Physician |
| 2020 | Amitai, Yona; Shental, Helen; Atkins-Manelis, Luba; Koren, Gideon; Zamir, Chen Stein | Pre-conceptional folic acid supplementation: A possible cause for the increasing rates of ankyloglossia | Medical Hypotheses |
| 2018 | Perez-Aguirre, Brenda; Soto-Barreras, Uriel; Loyola-Rodriguez, Juan Pablo; Reyes-Macias, Juan Francisco; Santos-Diaz, Miguel Angel; Loyola-Leyva, Alejandra; Garcia-Cortes, Obed | Oral findings and its association with prenatal and perinatal factors in newborns | Korean Journal of Pediatrics |
| 1998 | Houdayer, C.; Bahuau, M. | Orofacial cleft defects: inference from nature and nurture | Annales De Genetique |
| 2020 | Baxter, Richard; Merkel-Walsh, Robyn; Baxter, Barbara Stark; Lashley, Ashley; Rendell, Nicholas R. | Functional Improvements of Speech, Feeding, and Sleep After Lingual Frenectomy Tongue-Tie Release: A Prospective Cohort Study. | Clinical pediatrics |
| 2021 | Barberá-Pérez, Paula M.; Sierra-Colomina, Montserrat; Deyanova-Alyosheva, Nyulyufer; Plana-Fernández, Mariano; Lalaguna-Mallada, Paula | Prevalence of ankyloglossia in newborns and impact of frenotomy in a Baby-Friendly Hospital. | Boletin medico del Hospital Infantil de Mexico |
| 2021 | Shah, Shalini; Allen, Paul; Walker, Ryan; Rosen-Carole, Casey; McKenna Benoit, Margo K. | Upper Lip Tie: Anatomy, Effect on Breastfeeding, and Correlation With Ankyloglossia. | The Laryngoscope |
| 2019 | Schlatter, Sara-Maria; Schupp, Wiebke; Otten, Jörg-Elard; Harnisch, Sabine; Kunze, Mirjam; Stavropoulou, Dimitra; Hentschel, Roland | The role of tongue-tie in breastfeeding problems-A prospective observational study. | Acta paediatrica (Oslo, Norway : 1992) |
| 2021 | Slagter, Kirsten W.; Raghoebar, Gerry M.; Hamming, Inge; Meijer, Jiska; Vissink, Arjan | Effect of frenotomy on breastfeeding and reflux: results from the BRIEF prospective longitudinal cohort study. | Clinical oral investigations |
| 2021 | Maya-Enero, Silvia; Pérez-Pérez, Maria; Ruiz-Guzmán, Luis; Duran-Jordà, Xavier; López-Vílchez, María Ángeles | Prevalence of neonatal ankyloglossia in a tertiary care hospital in Spain: a transversal cross-sectional study. | European journal of pediatrics |
| 2015 | Ingram, Jenny; Johnson, Debbie; Copeland, Marion; Churchill, Cathy; Taylor, Hazel; Emond, Alan | The development of a tongue assessment tool to assist with tongue-tie identification. | Archives of disease in childhood. Fetal and neonatal edition |
| 2019 | de Oliveira, Alline Jesuino; Duarte, Danilo Antônio; Diniz, Michele Baffi | Oral Anomalies In Newborns: An Observational Cross-Sectional Study. | Journal of dentistry for children (Chicago, Ill.) |
| 2014 | Riskin, Arieh; Mansovsky, Michal; Coler-Botzer, Tzviya; Kugelman, Amir; Shaoul, Ron; Hemo, Miri; Wolff, Leslie; Harpaz, Sarit; Olchov, Zhana; Bader, David | Tongue-tie and breastfeeding in newborns-mothers' perspective. | Breastfeeding medicine : the official journal of the Academy of Breastfeeding Medicine |
| 2020 | França, Ellia Christinne Lima; Albuquerque, Lucas Carvalho Aragão; Martinelli, Roberta Lopes Castro; Gonçalves, Ilda Machado Fiuza; Souza, Cejana Baiocchi; Barbosa, Maria Alves | Surface Electromyographic Analysis of the Suprahyoid Muscles in Infants Based on Lingual Frenulum Attachment during Breastfeeding. | International journal of environmental research and public health |
| 2018 | Billington, Jennifer; Yardley, Iain; Upadhyaya, Manasvi | Long-term efficacy of a tongue tie service in improving breast feeding rates: A prospective study. | Journal of pediatric surgery |
| 2018 | Ghaheri, Bobak A.; Cole, Melissa; Mace, Jess C. | Revision Lingual Frenotomy Improves Patient-Reported Breastfeeding Outcomes: A Prospective Cohort Study. | Journal of human lactation : official journal of International Lactation Consultant Association |
| 2018 | Oakes, Tina Marie Myers; Skljarevski, Vladimir; Zhang, Qi; Kielbasa, William; Hodsdon, Michael E.; Detke, Holland C.; Camporeale, Angelo; Saper, Joel R. | Safety of galcanezumab in patients with episodic migraine: A randomized placebo-controlled dose-ranging Phase 2b study. | Cephalalgia : an international journal of headache |
| 2015 | Rose, Katie; Kasbekar, Anand V.; Flynn, Alison; De, Sujata | Developing a nurse-delivered frenulotomy service. | Otolaryngology--head and neck surgery : official journal of American Academy of Otolaryngology-Head and Neck Surgery |
| 2020 | Kim, Tae Hoon; Lee, Young Chan; Yoo, Seung Don; Lee, Seung Ah; Eun, Young-Gyu | Comparison of simple frenotomy with 4-flap Z-frenuloplasty in treatment for ankyloglossia with articulation difficulty: A prospective randomized study. | International journal of pediatric otorhinolaryngology |
| 2014 | González Jiménez, D.; Costa Romero, M.; Riaño Galán, I.; González Martínez, M. T.; Rodríguez Pando, M. C.; Lobete Prieto, C. | [Prevalence of ankyloglossia in newborns in Asturias (Spain)]. | Anales de pediatria (Barcelona, Spain : 2003) |
| 2012 | Berry, Janet; Griffiths, Mervyn; Westcott, Carolyn | A double-blind, randomized, controlled trial of tongue-tie division and its immediate effect on breastfeeding. | Breastfeeding medicine : the official journal of the Academy of Breastfeeding Medicine |
| 2021 | Mazzoni, Adriana; Navarro, Ricardo Scarparo; Fernandes, Kristianne Porta Santos; Horliana, Anna Carolina Ratto Tempestini; Mesquita-Ferrari, Raquel Agnelli; Motta, Pamella Barros; Silva, Tamiris; Gomes, Andréa Oliver; Martimbianco, Ana Luiza Cabrera; Sobral, Ana Paula Taboada; Santos, Elaine Marcilio; Motta, Lara Jansiski; Bussadori, Sandra Kalil | Evaluation of the effects of high-level laser and electrocautery in lingual frenectomy surgeries in infants: protocol for a blinded randomised controlled clinical trial. | BMJ open |
| 2015 | Sharma, S. D.; Jayaraj, S. | Tongue-tie division to treat breastfeeding difficulties: our experience. | The Journal of laryngology and otology |
| 2018 | Dixon, Bronwyn; Gray, Juliet; Elliot, Nikki; Shand, Brett; Lynn, Adrienne | A multifaceted programme to reduce the rate of tongue-tie release surgery in newborn infants: Observational study. | International journal of pediatric otorhinolaryngology |
| 2014 | Ovental, A.; Marom, R.; Botzer, E.; Batscha, N.; Dollberg, S. | Using topical benzocaine before lingual frenotomy did not reduce crying and should be discouraged. | Acta paediatrica (Oslo, Norway : 1992) |
| 2005 | Hogan, Monica; Westcott, Carolyn; Griffiths, Mervyn | Randomized, controlled trial of division of tongue-tie in infants with feeding problems. | Journal of paediatrics and child health |
| 2021 | Fioravanti, Miriam; Zara, Francesca; Vozza, Iole; Polimeni, Antonella; Sfasciotti, Gian Luca | The Efficacy of Lingual Laser Frenectomy in Pediatric OSAS: A Randomized Double-Blinded and Controlled Clinical Study. | International journal of environmental research and public health |
| 2017 | Shavit, Itai; Peri-Front, Yael; Rosen-Walther, Anda; Grunau, Ruth E.; Neuman, Gal; Nachmani, Omri; Koren, Gideon; Aizenbud, Dror | A Randomized Trial to Evaluate the Effect of Two Topical Anesthetics on Pain Response During Frenotomy in Young Infants. | Pain medicine (Malden, Mass.) |
| 2004 | Griffiths, D. Mervyn | Do tongue ties affect breastfeeding? | Journal of human lactation : official journal of International Lactation Consultant Association |
| 2014 | Emond, Alan; Ingram, Jenny; Johnson, Debbie; Blair, Peter; Whitelaw, Andrew; Copeland, Marion; Sutcliffe, Alastair | Randomised controlled trial of early frenotomy in breastfed infants with mild-moderate tongue-tie. | Archives of disease in childhood. Fetal and neonatal edition |
| 2006 | Dollberg, Shaul; Botzer, Eyal; Grunis, Esther; Mimouni, Francis B. | Immediate nipple pain relief after frenotomy in breast-fed infants with ankyloglossia: a randomized, prospective study. | Journal of pediatric surgery |
| 1976 | Yoel, J. | [Tongue tie and speech disorders]. | La Tribuna odontologica |
| 2002 | Kato, Junji; Jayawardena, Jayanetti Asiri; Wijeyeweera, Rafel Luxhmen; Moriya, Kayoko; Takagi, Yuzo | [Application of a CO₂ laser for oral soft tissue surgery in children in Sri Lanka--introduction of a laser through activities of aid to a developing country]. | Kokubyo Gakkai zasshi. The Journal of the Stomatological Society, Japan |
| 2020 | Diercks, Gillian R.; Hersh, Cheryl J.; Baars, Rebecca; Sally, Sarah; Caloway, Christen; Hartnick, Christopher J. | Factors associated with frenotomy after a multidisciplinary assessment of infants with breastfeeding difficulties. | International journal of pediatric otorhinolaryngology |
| 2021 | Shang, Jiang; Han, Maoqiang; Sun, Jing; Xu, Dapeng; Qu, Weidong | Comparative Study on the Treatment of Ankyloglossia by Using Er:YAG Laser or Traditional Scalpel. | The Journal of craniofacial surgery |
| 2011 | Buryk, Melissa; Bloom, David; Shope, Timothy | Efficacy of neonatal release of ankyloglossia: a randomized trial. | Pediatrics |
| 2022 | Carminatti, MÔnica; Nicoloso, Gabriel Ferreira; Miranda, Priscilla Polinesi; Gomes, Erissandra; de Araujo, Fernando Borba | The Effectiveness of Lingual Frenectomy and Myofunctional Therapy for Children: A Randomized Controlled Clinical Trial. | Journal of dentistry for children (Chicago, Ill.) |
| 2021 | Varadarajan, S.; Balaji, T.M.; Raj, A.T.; Gupta, A.A.; Patil, S.; Alhazmi, T.H.; Alaqi, H.A.A.; Al Omar, N.E.M.; Almutaher, S.A.B.A.; Jafer, A.A.; Hedad, I.A. | Genetic Mutations Associated with Pierre Robin Syndrome/Sequence: A Systematic Review | Molecular Syndromology |
| 2021 | Nasreddine, G.; El Hajj, J.; Ghassibe-Sabbagh, M. | Orofacial clefts embryology, classification, epidemiology, and genetics | Mutation Research - Reviews in Mutation Research |
| 2020 | Martinelli, M.; Palmieri, A.; Carinci, F.; Scapoli, L. | Non-syndromic Cleft Palate: An Overview on Human Genetic and Environmental Risk Factors | Frontiers in Cell and Developmental Biology |
| 2020 | Paolini, S.L.; Pilato, M.; Rajasekaran, V.; Waters, J.F.R.; Bagic, A.; Urban, A. | Outcomes in three cases after brivaracetam treatment during pregnancy | Acta Neurologica Scandinavica |
| 2020 | Sedley, L. | Advances in Nutritional Epigenetics—A Fresh Perspective for an Old Idea. Lessons Learned, Limitations, and Future Directions | Epigenetics Insights |
| 2018 | Clark, M.B.; Clark, D.A. | Oral development and pathology | Ochsner Journal |
| 2017 | Figueiredo, R.F.; Figueiredo, N.; Feguri, A. | The main epidemiological aspects of folic acid and the prevention of orofacial clefts |  |
| 2017 | Tettamanti, L.; Avantaggiato, A.; Nardone, M.; Palmieri, A.; Tagliabue, A. | New insights in orofacial cleft: Epidemiological and genetic studies on Italian samples | ORAL and Implantology |
| 2016 | Tirado Amador, L.R.; Anaya, M.V.M.; González Martínez, F.D. | Genetic and epigenetic interactions related to non-syndromic cleft lip and palate [Interacciones genéticas y epigenéticas relacionadas con fisuras de labio y paladar no sindrómicas] | Avances en Odontoestomatologia |
| 2015 | Fu, X.; Cheng, Y.; Yuan, J.; Huang, C.; Cheng, H.; Zhou, R. | Loss-of-function mutation in the X-linked TBX22 promoter disrupts an ETS-1 binding site and leads to cleft palate | Human Genetics |
| 2015 | Bilińska, M.; Osmola, K. | Cleft lip and palate - risk factors, prenatal diagnosis, and health consequences [Rozszczep wargi i podniebienia - czynniki ryzyka, diagnostyka prenatalna i konsekwencje zdrowotne] | Ginekologia Polska |
| 2014 | Lin, Y.; Shu, S.; Tang, S. | A case-control study of environmental exposures for nonsyndromic cleft of the lip and/or palate in eastern Guangdong, China | International Journal of Pediatric Otorhinolaryngology |
| 2013 | Carlson, B.M. | Human Embryology and Developmental Biology: Fifth Edition |  |
| 2012 | Kohli, S.S.; Kohli, V.S. | A comprehensive review of the genetic basis of cleft lip and palate | Journal of Oral and Maxillofacial Pathology |
| 2012 | Rahimov, F.; Jugessur, A.; Murray, J.C. | Genetics of nonsyndromic orofacial clefts | Cleft Palate-Craniofacial Journal |
| 2011 | Dixon, M.J.; Marazita, M.L.; Beaty, T.H.; Murray, J.C. | Cleft lip and palate: Understanding genetic and environmental influences | Nature Reviews Genetics |
| 2010 | Scully, C.; Hegarty, A. | The Oral Cavity and Lips |  |
| 2010 | Morriss-Kay, G.M. | The Head |  |
| 2009 | Yu, W.; Serrano, M.; Miguel, S.S.; Ruest, L.B.; Svoboda, K.H. | Cleft lip and palate genetics and application in early embryological development | Indian Journal of Plastic Surgery |
| 2009 | Mossey, P.A.; Little, J.; Munger, R.G.; Dixon, M.J.; Shaw, W.C. | Cleft lip and palate | The Lancet |
| 2009 | Murthy, J.; Bhaskar, L.V.K.S. | Current concepts in genetics of nonsyndromic clefts | Indian Journal of Plastic Surgery |
| 2006 | Krapels, I.P.; Vermeij-Keers, C.; Müller, M.; De Klein, A.; Steegers-Theunissen, R.P. | Nutrition and genes in the development of orofacial clefting | Nutrition Reviews |
| 2006 | Aronson, J.K. | Meyler’s side effects of drugs: The international encyclopedia of adverse drug reactions and interactions |  |
| 2005 | Castriota-Scanderbeg, A.; Dallapiccola, B. | Abnormal skeletal phenotypes: From simple signs to complex diagnoses |  |
| 2005 | Lawrence, R.A.; Lawrence, R.M. | Breastfeeding |  |
| 2004 | Martinelli, M.; Carinci, F.; Scapoli, L.; Pezzetti, F.; Marchesini, J.; Palmieri, A.; Caramelli, E.; Baciliero, U.; Padula, E.; Gombos, F.; Rullo, R.; Carls, F.; Becchetti, A.; Tognon, M.; Carinci, P. | Drugs, environmental factors, loci and genes involved in nonsyndromic orofacial cleft | Current Pharmacogenomics |
| 2003 | Batra, P.; Duggal, R.; Parkash, H. | Genetics of cleft lip and palate revisited | Journal of Clinical Pediatric Dentistry |
| 2003 | Carinci, F.; Pezzetti, F.; Scapoli, L.; Martinelli, M.; Avantaggiato, A.; Carinci, P.; Padula, E.; Baciliero, U.; Gombos, F.; Laino, G.; Rullo, R.; Cenzi, R.; Carls, F.; Tognon, M. | Recent Developments in Orofacial Cleft Genetics | Journal of Craniofacial Surgery |
| 2002 | Murray, J.C. | Gene/environment causes of cleft lip and/or palate | Clinical Genetics |
| 2001 | Spritz, R.A. | The genetics and epigenetics of orofacial clefts | Current Opinion in Pediatrics |
| 2000 | Prescott, N.J.; Lees, M.M.; Winter, R.M.; Malcolm, S. | Identification of susceptibility loci for nonsyndromic: Cleft lip with or without cleft palate in a two stage genome scan of affected sib-pairs | Human Genetics |
| 2015 | Suchdev, , PS, Peña‐Rosas, JP; De‐Regil, LM | Multiple micronutrient powders for home (point‐of‐use) fortification of foods in pregnant women | Cochrane Database of Systematic Reviews |
| 2013 | Lassi, , ZS, Salam, RA, Haider, BA; Bhutta, ZA | Folic acid supplementation during pregnancy for maternal health and pregnancy outcomes | Cochrane Database of Systematic Reviews |
| 2015 | Peña‐Rosas, , JP, De‐Regil, LM, Garcia‐Casal, MN; Dowswell, T | Daily oral iron supplementation during pregnancy | Cochrane Database of Systematic Reviews |
| 2020 | Baxter, Richard; Merkel-Walsh, Robyn; Baxter, Barbara Stark; Lashley, Ashley; Rendell, Nicholas R. | Functional Improvements of Speech, Feeding, and Sleep After Lingual Frenectomy Tongue-Tie Release: A Prospective Cohort Study | Clinical Pediatrics |

**Table S1: Included/Excluded Articles**

| Year | Author | Title | Journal |
| --- | --- | --- | --- |
| 2010 | Reamy, Brian V.; Derby, Richard; Bunt, Christopher W. | Common tongue conditions in primary care | American Family Physician |
| 2020 | Amitai, Yona; Shental, Helen; Atkins-Manelis, Luba; Koren, Gideon; Zamir, Chen Stein | Pre-conceptional folic acid supplementation: A possible cause for the increasing rates of ankyloglossia | Medical Hypotheses |
| 2018 | Perez-Aguirre, Brenda; Soto-Barreras, Uriel; Loyola-Rodriguez, Juan Pablo; Reyes-Macias, Juan Francisco; Santos-Diaz, Miguel Angel; Loyola-Leyva, Alejandra; Garcia-Cortes, Obed | Oral findings and its association with prenatal and perinatal factors in newborns | Korean Journal of Pediatrics |
| 2021 | Barberá-Pérez, Paula M.; Sierra-Colomina, Montserrat; Deyanova-Alyosheva, Nyulyufer; Plana-Fernández, Mariano; Lalaguna-Mallada, Paula | Prevalence of ankyloglossia in newborns and impact of frenotomy in a Baby-Friendly Hospital. | Boletin medico del Hospital Infantil de Mexico |
| 2021 | Shah, Shalini; Allen, Paul; Walker, Ryan; Rosen-Carole, Casey; McKenna Benoit, Margo K. | Upper Lip Tie: Anatomy, Effect on Breastfeeding, and Correlation With Ankyloglossia. | The Laryngoscope |
| 2019 | Schlatter, Sara-Maria; Schupp, Wiebke; Otten, Jörg-Elard; Harnisch, Sabine; Kunze, Mirjam; Stavropoulou, Dimitra; Hentschel, Roland | The role of tongue-tie in breastfeeding problems-A prospective observational study. | Acta paediatrica (Oslo, Norway : 1992) |
| 2021 | Maya-Enero, Silvia; Pérez-Pérez, Maria; Ruiz-Guzmán, Luis; Duran-Jordà, Xavier; López-Vílchez, María Ángeles | Prevalence of neonatal ankyloglossia in a tertiary care hospital in Spain: a transversal cross-sectional study. | European journal of pediatrics |
| 2019 | de Oliveira, Alline Jesuino; Duarte, Danilo Antônio; Diniz, Michele Baffi | Oral Anomalies In Newborns: An Observational Cross-Sectional Study. | Journal of dentistry for children (Chicago, Ill.) |
| 2014 | Riskin, Arieh; Mansovsky, Michal; Coler-Botzer, Tzviya; Kugelman, Amir; Shaoul, Ron; Hemo, Miri; Wolff, Leslie; Harpaz, Sarit; Olchov, Zhana; Bader, David | Tongue-tie and breastfeeding in newborns-mothers' perspective. | Breastfeeding medicine : the official journal of the Academy of Breastfeeding Medicine |
| 2014 | González Jiménez, D.; Costa Romero, M.; Riaño Galán, I.; González Martínez, M. T.; Rodríguez Pando, M. C.; Lobete Prieto, C. | [Prevalence of ankyloglossia in newborns in Asturias (Spain)]. | Anales de pediatria (Barcelona, Spain : 2003) |
| 2012 | Berry, Janet; Griffiths, Mervyn; Westcott, Carolyn | A double-blind, randomized, controlled trial of tongue-tie division and its immediate effect on breastfeeding. | Breastfeeding medicine : the official journal of the Academy of Breastfeeding Medicine |
| 2015 | Sharma, S. D.; Jayaraj, S. | Tongue-tie division to treat breastfeeding difficulties: our experience. | The Journal of laryngology and otology |
| 2018 | Dixon, Bronwyn; Gray, Juliet; Elliot, Nikki; Shand, Brett; Lynn, Adrienne | A multifaceted programme to reduce the rate of tongue-tie release surgery in newborn infants: Observational study. | International journal of pediatric otorhinolaryngology |
| 2005 | Hogan, Monica; Westcott, Carolyn; Griffiths, Mervyn | Randomized, controlled trial of division of tongue-tie in infants with feeding problems. | Journal of paediatrics and child health |
| 2004 | Griffiths, D. Mervyn | Do tongue ties affect breastfeeding? | Journal of human lactation : official journal of International Lactation Consultant Association |
| 2014 | Emond, Alan; Ingram, Jenny; Johnson, Debbie; Blair, Peter; Whitelaw, Andrew; Copeland, Marion; Sutcliffe, Alastair | Randomised controlled trial of early frenotomy in breastfed infants with mild-moderate tongue-tie. | Archives of disease in childhood. Fetal and neonatal edition |
| 2006 | Dollberg, Shaul; Botzer, Eyal; Grunis, Esther; Mimouni, Francis B. | Immediate nipple pain relief after frenotomy in breast-fed infants with ankyloglossia: a randomized, prospective study. | Journal of pediatric surgery |
| 2020 | Diercks, Gillian R.; Hersh, Cheryl J.; Baars, Rebecca; Sally, Sarah; Caloway, Christen; Hartnick, Christopher J. | Factors associated with frenotomy after a multidisciplinary assessment of infants with breastfeeding difficulties. | International journal of pediatric otorhinolaryngology |
| 2011 | Buryk, Melissa; Bloom, David; Shope, Timothy | Efficacy of neonatal release of ankyloglossia: a randomized trial. | Pediatrics |
| 2020 | Sedley, L. | Advances in Nutritional Epigenetics—A Fresh Perspective for an Old Idea. Lessons Learned, Limitations, and Future Directions | Epigenetics Insights |
| 2017 | Figueiredo, R.F.; Figueiredo, N.; Feguri, A. | The main epidemiological aspects of folic acid a  nd the prevention of orofacial clefts |  |
| 2015 | Suchdev, , PS, Peña‐Rosas, JP; De‐Regil, LM | Multiple micronutrient powders for home (point‐of‐use) fortification of foods in pregnant women | Cochrane Database of Systematic Reviews |
| 2013 | Lassi, , ZS, Salam, RA, Haider, BA; Bhutta, ZA | Folic acid supplementation during pregnancy for maternal health and pregnancy outcomes | Cochrane Database of Systematic Reviews |
| 2015 | Peña‐Rosas, , JP, De‐Regil, LM, Garcia‐Casal, MN; Dowswell, T | Daily oral iron supplementation during pregnancy | Cochrane Database of Systematic Reviews |
| 2007 | Segal, Lauren M.; Stephenson, Randolph; Dawes, Martin; Feldman, Perle | Prevalence, diagnosis, and treatment of ankyloglossia | Canadian Family Physician |

**S2 Appendix: PROSPERO Registration**

PROSPERO Registration ID: CRD42022375862, Registered 01.12.2022
